# Neoplastic Bone Marrow Niche: Hematopoietic and Mesenchymal Stem Cells

**Published:** 2011-09-23

**Authors:** Najmaldin Saki, Saeid Abroun, Majid Farshdousti Hagh, Farahnaz Asgharei

**Affiliations:** 1. Department of Hematology and Blood Banking School of Medical Sciences, Tarbiat Modares University, Tehran, Iran; 2. Research Center of Thalassemia and Hemoglobinopathy, Ahvaz Jundishapur University of Medical Sciences, Ahvaz, Iran; 3. Department of Hematology, Tabriz University of Medical Sciences, Tabriz, Iran; 4. Depatment of Immunogenetic, Rostok University, Rostok, Germany

**Keywords:** Neoplastic Niche, Stem Cells, Bone Marrow, Cancer

## Abstract

The neoplastic niche comprises complex interactions between multiple cell types and molecules requiring cell-cell signaling as well as local secretion. These niches are important for both the maintenance of cancer stem cells and the induction of neoplastic cells survival and proliferation. Each niche contains a population of tumor stem cells supported by a closely associated vascular bed comprising mesenchyme-derived cells and extracellular matrix. Targeting cancer stem cells and neoplastic niche may provide new therapies to eradicate tumors. Much progress has been very recently made in the understanding of the cellular and molecular interactions in the microenvironment of neoplastic niches. This review article provides an overview of the neoplastic niches in the bone marrow. In addition to highlighting recent advances in the field, we will also discuss components of the niche and their signaling pathways.

## Introduction

 Bone marrow microenvironment contains stem cells and other cells that are supported by hematopoietic and mesenchymal stem cells (MSCs). These cells can differentiate into several cell lineages (Table 1). Osteoclasts and osteoblasts play important role in microenvironment destruction and construction(1). Osteoblasts derive from MSCs that play an important role in the construction of Osteoblastic/endosteal niches. Osteoblastic niches maintain hematopoietic stem cells (HSCs) in quiescence phase (2). Cancer initiating cells disrupt cell-cell and cell-matrix interactions in the bone marrow. Therefore, they can change intact niches into neoplastic niches. Therefore,the bone marrow microenvironment plays an important role in the fate determination of HSCs and MSCs in several biological process (including apoptosis, self-renewal, expansion and differentiation)(3, 4).

**Table 1:** Cell-derived hematopoietic and mesenchymal stem cells


Hematopoietic stem cells	Common lymphoid progenitor	B Cells	
		T Cells	
		NK Cells	
	Common myeloid progenitor	Granulocyte-Macrophage progenitors	Granulocyte
			Monocyte
			Macrophage
			Osteoclast
		Megakaryocyte-Erythroid progenitors	Megakaryocyte
			Erythrocyte

Mesenchymal stem cells	Chondrocyte, Osteoblast, Fibroblast, Adipocyte, Myocyte,Endothelial cells		


The factors secreted in niche including chemokines, cytokines and growth factors have significant effect on the other residing cells in the bone marrow. Induced signaling by these factors can affect the fate of stem cells and other cells(5, 6). Therefore, knowing the effective mechanisms and molecules inducing neoplastic niche can help in targeted treatment of hematological diseases.

 Bone marrow is the main source of MSCs (p<0.05%). MSCs are mononuclear, adhering cells having the capacity for self-renewal, supporting hematopoiesis and the ability to differentiate into several cell lineages (e.g. chondrocytes, osteoblasts, adipocytes, dopaminergic neurons, astrocytes, tenocytes and endothelial cells)(7-10). They express specific and nonspecific surface markers (Table 2).

 Also other identifying markers such as D7Fib, STRO-1 and SSEA exist on MSCs (11, 12). MSCs are devoid of hematopoietic and endothelial markers (e.g. CD11b, CD14, CD31, CD45) and co-stimulatory markers (e.g. CD80 and CD81) (13). Rat MSCs express Thy-1 and c-Kit (stem cell markers), VWF (endothelial cells marker) and a-smooth muscle actin (smooth muscle cells marker) (14).

MSCs secrete several biological factors such as anti-apoptotic factors [including vascular endothelial growth factor (VEGF), hepatocyte growth factor (HGF), insulin-like growth factor (IGF-1), basic fibroblast growth factor (bFGF) and granulocyte/macrophage colony stimulating factor (GM-CSF)], immunomodulatory factors (including PGE2, TGF-β, IDO, iNOS anf LIF) and other factors including SCF, IL6, Angiopoietin-1, IL8 and SDF1/CXCL12, IL1, tumor necrosis factor-α (TNF-α) and bone morphogenetic proteins (BMPs) (15, 16). MSCs inhibit differentiation and activation of dendritic cells by producing PGE2, M-CSF, IL6, IL10 and TGF-β (13). Differentiation of hBMSCs into endothelial like-cells was induced in the presence of VEGF and IGF-1 (10, 17). They were also transdifferentiate into cardiomyocyte-like cells by 5-azacytidine and VEGF (18). Dexamethasone promotes the differentiation of MSCs into osteoblasts in vitro. Receptor activator of nuclear factor kappa-B ligand (RANKL) and osteoprotegerin (OPG) are expressed by MSCs. Some studies have indicated that expression of OPG and RANKL respectively, is decreased and increased in MSCs with ageing (19). HSCs and MSCs synthesize BMPs. Both BMP6 and BMP2 increase osteoblastic differentiation anddecrease differentiation of HSCs towards osteoclasts (20). Retinoid signaling may synergize with BMP9 activity in promoting the osteogenic differentiation of MPCs (21). Several studies have revealed that there is a balance between Wnt, FGFR and BMP signaling. Because BMP and Wnt signaling pathway lead to chondrocyte differentiation while EGF ends up to osteoblasts differentiation (22). Insulin like growth factor-1 plays a role in overexpression of CXCR4, leading to the enhancement of SDF-1 induced MSCs migration (10). Also IGF1 has a role in the mesenchymal and osteoprogenitor cells recruitment and proliferation (23). MSCs exhibit the capacity to promote engraftment of CD34+ UCB cells in irradiated mice, because MSCs produce some essential hematopoietic growth factors(24). MSCs secrete dickkopf-1 (DKK-1), a Wnt signaling pathway antagonist. Wnt signaling plays a vital role in both the self-renewal and differentiation of stem cells (25). DKK-1 may act as an autocrine inhibitor.

The differentiation of MSCs into osteoblastic lineage is regulated by the BMPs and Wnt signaling (26). MSCs express RUNX2, a transcription factor that is stimulated by FGFR2 signaling and is an essential transcription factor in osteoblastogenesis (27). Wnt signaling promotes the osteoblast differentiation and function. Former studies have shown that binding of FGF to FGFR2 results in the induction of Sox2 expression, and leads to blocking the osteoblastic differentiation of MSCs and Wnt signaling. This inhibition results from the interference of Sox2 with β-Catenin and numerous other Wnt target genes such as Wisp1, Wisp2, Dlx2, and msx2 (28, 29). Overexpression of miR-200a in Mesenchymal-like c666-1 cells inhibits β-Catenin signaling (30). TLR2, TLR4, NOD1 and NOD2 genes are expressed in hUCB-MSCs. Both NLRs and TLRs may be involved in the osteogenic differentiation of MSCs because some TLR and NLR agonists increase osteogenic differentiation of hUCB- MSCs (31). Transcription factors such as Cbfa1/RUNX2, Msx2, Dlx5 and SP7 are activated during the osteogenic differentiation of MSCs. Osteoblastic transcription factors induce the expression of bone-related marker genes including SPP1, COLIA1, SPARC and osteocalcin during the osteogenic differentiation (27). ERK phosphorylation is correlated with the osteogenic differentiation of MSCs (31). Notch maintains Mesenchymal precursor cells in an undifferentiated state. Notch1 overexpression under the control of the Prx1 enhancer induced Mesenchymal precursor cells proliferation and suppresses their differentiation (26). Peroxisome proliferator-activated receptor-gamma (PPAR-λ) is a key regulator of the adipogenesis whereas hedgehog signaling inhibits adipogenesis and induces osteoblastogenesis in MSCs. IL-1, TNF-α and noncanonical Wnt signaling pathway suppress the PPAR-λ function in MSCs (32).

**Table 2:** The CD markers of Mesenchymal stem cells


Marker	Name	Chromosome	Cellular expression	Role	Expression increased by	References
CD9	Motility related protein 1 (MRP1)	12p13.3	B1, MZ, pre-B cell	Cell adhesion, proliferation, motility, osteoclastogenesis, metastasis supresor, signal transduction	SDF1	33	
CD13	Aminopeptidase N (APN)	15q25-q26	Myeloid cells, Fibroblasts, osteoclasts, endothelial cells, BM stromal cells, Neuronals synaptic mem- branes	Presenting antigens, regulatory role in T cells, angiogenesis	TGFβ1, c-Maf	34	
CD29	Integrin β1	10p11.2	Th17, fibroblasts, monocytes, mast cells, endothelial cells	Cell-cell and cell-matrix interaction, adhesion	-	34-38	
CD44	H-CAM	11p13	-	Cell adhesion and migration, secration and activation of MMP-2, T cell acumulation	-	17,33, 34	
CD49e	Integrin α5 (VLA-5)	12q11-q13	T cells, B cells, monocytes	Cell migration, development and survival, signal trans- duction (RTK/Ras/Erk)	-	34	
CD54	ICAM1	19p13.3-p13.2	Leukocytes	Innate and adaptive immune, tumor cell growth arrest	-	33,34	
CD55	Decay-accelerating factor (DAF)	1q32	Many cell types(RBC,WBC)	Inhibition of complement activation	-	33	
CD71	Transferrin receptor	3q29	Erythroid lineage	Iron uptake, Erk-MAPK signaling	-	33	
CD73	5-terminal nucleotidase	6q14-q21	B cells and T cells, lymphatic vessels	Prevent TRALI-induced apoptosis, suppression of pro-inflammatory process	HIF-1	33, 34	
CD90	Thy1 glycoprotein	11q23.3	Neurons, fibroblasts	Apoptotic signaling, tumor suppression , Thy1 signaling	Iron level	33, 35	
CD105	Endoglin	9q33-q34.1	Endothelial cells, monocytes	Angiogenesis, TGFβ/ ALK1 signaling, anti-apoptotic effect	Hypoxia, TGFβ1	17, 33, 34	
CD123	IL3RA	Xp22.3 or Yp11.3	Plasmacytoid dendritic cells	STAT5 and c-fos activation	-	33	
CD124	IL4R	16p12.1-p11.2	B and T cells	IL4Rα signaling, immune modulation	-	33	
CD126	IL6R	1q21	Hepatocytes, neutrophils, monocytes/macrophage, lymphocytes	Signal transduction, JAK1/ Tyk2 and Ras activation	-	33	
CD127	IL7R	5p13	NaÏve T cells, granulosa cells, cumulus cells, preovulatory oocytes	T cell survival, upregulation of Bcl_2_ and Glut1	Foxo1	33	
CD166	ALCAM (Activated leukocyte cell adhesion molecule)	3q13.1	Neurons, T cells, monocytes	-	-	17, 33, 34	
ASMA	α-smooth muscle actin	10q23.3	-	-	-	34	
CD271	Low affinty nerve growth factor receptor (LNGFR)	17q21-q22	-	-	-	39	


### Hematopoietic stem cells

HSCs express markers such as CD34, CD45 and CD133 and have properties such as clonogenicity, self-renewal, and multipotentiality(40). combination of surface markers of the signaling lymphocyte activation molecule (SLAM) family(CD150^+^CD48^−^CD41^−^) was shown to be an HSC identifier (2). However, another study has recently shown that SLAM family markers do not permit the same degree of HSC enrichment in humans and rhesus macaques as in mice (41). Activity of osteoblasts and osteoclasts is coordinately regulated. Osteoblasts impact osteoclasts development, which is derived from HSCs (42). Osteoclasts functions are fundamental in the HSC niche (43). The factors secreted by stromal cells (e.g. heparan sulfate) have the capacity to support HSCs (44).

Notch receptors and their ligands are involved in HSCs maintenance(45). Notch1 (N1) in the murine bone marrow progenitors increases the HSC-self-renewal and immortalization of stem cells like progenitors (46). Several studies have recently shown that osteoblasts expressing the Notch ligand Jagged1 (J1) were identified as being part of the HSC niche (47, 48). Expression of the parathyroid hormone related protein (PTHrP) receptor in osteoblasts brings about increased numbers of osteoblasts to express high levels of J1 (48). Therefore,increased number of osteoblasts is accompaniedwith an increase in the number of HSCs, resulting in Notch1 activation in HSCs and following self-renewality of HSCs (48). These results show that J1-expressing osteoblasts regulate HSCs self renewality through Notch1 activation. However,Manicini et al. showed that HSCs self-renewality and differentiation are independent of Jagged1 dependent Notch signaling (49).

### Normal and neoplastic niche

Bone microenvironment consists of different cells such as stromal (osteoblasts, adipocytes, fibroblasts and endothelial cells) and non-stromal cells.Osteoclasts and osteoblasts have vital role in bone remodeling and niche structure (50, 51). Osteoblasts by expressing and secreting some molecules and factors regulate maturation and proliferation of other bone marrow resident cells, respectively.Osteoblasts regulate HSCs quiescence through the activation of Tie-2/Ang-1 signaling pathway (52).N-cadherin, a cell adhesion molecule, is expressed in both the osteoblasts and HSCs, suggesting that binding of N-cadherin induces the adhesion of HSCs to the niche cells (53). Studies have demonstrated that N-cad expression is down-regulated by reactive oxygen species (ROS), resulting in the release of HSCs from the niche cells. Therefore, N-cad-mediated cell adhesion has an important role in the maintaining and mobilizing of HSCs in bone marrow (54).

Osteoblasts regulate osteoclast maturation and proliferation by several factors such as RANKL (55). RANKL represents the essential osteoblastderived factor required for osteoclast formation and activation, and induces bone loss, whereas OPG blocks these effects and prevents bone resorption (56). OPG, a member of the tumor necrosis factor receptor (TNFR) superfamily, is secreted by osteoblastic lineage cells (57), and acts as a soluble receptor antagonist for RANKL (58). The expression of RANKL and OPG by osteoblastic lineage cells is modulated by a variety of osteotropic hormones and cytokines (56).

PTH enhances osteoblast function and also increases osteoclasts activity via the PTH/PTHrP receptor-1. Strontium (Sr) enhances osteoblast activity but inhibits osteoclasts by calcium sensing receptor. Sr increases the expression of Runx2, which is an essential transcription factor for osteoblastogenesis (59).

Normally, the stem cell niche maintains a balance between the quiescence and proliferation of stem cells(60). Wnt/beta-catenin growth promoting signals are counterbalanced by BMP anti-growth signals. If the dynamic balance is egregiously altered, then the dominant signalings such as Wnt/ beta-catenin activation or loss of BMP can lead to cancer formation within the hematopoietic, epidermal and gastrointestinal systems (61-63).

VEGFR1-positive hematopoietic progenitors cells (HPCs) initiate the pre-metastatic niche cells. HPCs migrate from the niches within the bone marrow to the primary tumor neovasculature and pre-metastatic niche cells. Bone marrow-derived cells lie quiescent in the osteoblastic niche, but when activated, move to the vascular niche from where they egress and home to the periphery sites. At the primary tumor site, they are essential for stability of newly forming vessels, and at the pre- metastatic niche cells, angiogenic elements are required for the establishment of metastatic lesion. VEGFR1-positive HPC form clusters in these sites prior to the arrival of tumor cells (64).

Cancer stem cells (CSCs) in bone marrow reside in both the vascular and osteoblastic niches. In the vascular niche, cross-talk occurs between the CSCs and endothelial cells, while in the osteoblastic niche, CSCs interact with both the osteoclasts and osteoblasts. Within both niches, MSCs influence tumor growth and CSCs inhibit normal HSCs. Bone marrow microenvironment plays a primary role in the hematologic malignancy pathophysiology, and recent evidence indicates that numbers of cytokines such as leptin in multiple myeloma enhance tumor cells growth in bone marrow microenvironment (65).

## Conclusion

Normal stem cells (NSCs) and CSCs are vital in tissue maintenance and malignant progression, respectively. The normal and neoplastic niches in which these cells reside play an indispensible role in NSCs and CSCs survival, proliferation, and differentiation. While the niches are each unique,there are many pathways and processes that appear to be conserved among these microenvironments. The interaction of the CSCs and the niche is a promising therapeutic target, and several drugs that affect neoplastic niche cross-talk are currently being investigated in clinical trials. Additional research is needed to further understand this complex neoplastic niche in order to fully realize the potential of this unique therapeutic target.
